# Towards a tailored indoor horticulture: a functional genomics guided phenotypic approach

**DOI:** 10.1038/s41438-018-0065-7

**Published:** 2018-11-01

**Authors:** Claudius Marondedze, Xinyun Liu, Shihui Huang, Cynthia Wong, Xuan Zhou, Xutong Pan, Huiting An, Nuo Xu, Xuechen Tian, Aloysius Wong

**Affiliations:** 1 Laboratoire de Physiologie Cellulaire et Végétale, Université Grenoble Alpes, CEA/DRF/BIG, INRA UMR1417, CNRS UMR5168, 38054 Grenoble Cedex 9, France; 2 Department of Biology, Wenzhou-Kean University, 88 Daxue Road, Ouhai, Wenzhou, Zhejiang Province 325060 China; 3 Department of Cell and Systems Biology, University of Toronto, 25 Willcocks Street, Toronto, ON M5S 3B2 Canada

## Abstract

As indoor horticulture gathers momentum, electric (also termed artificial) lighting systems with the ability to generate specific and tunable wavelengths have been developed and applied. While the effects of light quality on plant growth and development have been studied, authoritative and reliable sets of light formulae tailored for the cultivation of economically important plants and plant traits are lacking as light qualities employed across laboratories are inconsistent. This is due, at least in part, to the lack of molecular data for plants examined under electric lights in indoor environments. It has hampered progress in the field of indoor horticulture, in particular, the transition from small-scale indoor farming to commercial plant factories. Here, we review the effects of light quality on model and crop plants studied from a physiological, physical and biochemical perspective, and explain how functional genomics can be employed in tandem to generate a wealth of molecular data specific for plants cultivated under indoor lighting. We also review the current state of lighting technologies in indoor horticulture specifically discussing how recent narrow-bandwidth lighting technologies can be tailored to cultivate economically valuable plant species and traits. Knowledge gained from a complementary phenotypic and functional genomics approach can be harvested not only for economical gains but also for sustainable food production. We believe that this review serves as a platform that guides future light-related plant research.

## Indoor horticulture: Lighting the way to sustainability

Tailored multidisciplinary approaches to hone sustainable indoor horticulture could significantly improve plant yields and crop quality. Advances in artificial lighting systems could transform commercial-scale indoor horticulture, but the current technology is limited by a lack of molecular data for plants grown under such lighting schemes. Aloysius Wong at Wenzhou-Kean University in Wenzhou, China, and co-workers reviewed research into the effects of light quality and differing wavelengths on plant growth. The team advocate the use of plant type-specific and functional genomics studies to examine light-determined molecular traits and associated gene expression. These could be used to build an extensive catalog of light qualities that enhance indoor crop yields and quality. Combining LED lights of different colors and wavelengths shows promise, and the researchers highlight the potential of tunable narrow wavelength lights, such as lasers.

## Electric lighting has a growing influence in indoor horticulture

By 2050, the world population is projected to reach 9 billion and this will demand at least 70% more food to feed the growing population^1^. In an effort to fight hunger and malnutrition, people around the world face common interrelated challenges of population growth, resource availability, and environmental changes. As more cropland is devoted to non-food purposes such as fuel extraction, cotton farming, housing and other infrastructures, cropland expansion is no longer an attractive nor a feasible option^2^. Furthermore, environmental change including but not limited to the melting of glaciers, soil erosion, and desertification, threaten to reduce land productivity^3,4^. Thus, it is necessary to find sustainable means for growing plants especially food crops. One solution is indoor horticulture where electric light sources are employed in place of sunlight to grow plants in highly controlled closed environments where parameters (e.g., light, temperature, nutrient, carbon dioxide level, and humidity) essential for plant growth can be optimized and maintained throughout its development by a technology-driven approach. This approach enables careful management of waste emission and allows for the application of ‘‘vertical farming.’’ Vertical farming refers to the growing of crops in layers or inclined surfaces stacked vertically either on its own or integrated into structures with other primary functions such as skyscrapers and warehouses. This form of farming generates higher yield per unit area of land, thus providing attractive features especially for urban horticulture^5,6^. Resources such as water, light, and nutrients can be tightly regulated to optimize growth parameters yield within a clean enclosed area of farming that reduces the use of pesticides^7^. As such, indoor horticulture presents a sustainable approach to feed the growing population while minimizing the negative impact of agriculture on the environment. For instance, vertical farming has been previously shown to increase lettuce yield per unit area compared to conventional horizontal hydroponics^8^.

**Fig. 1:**
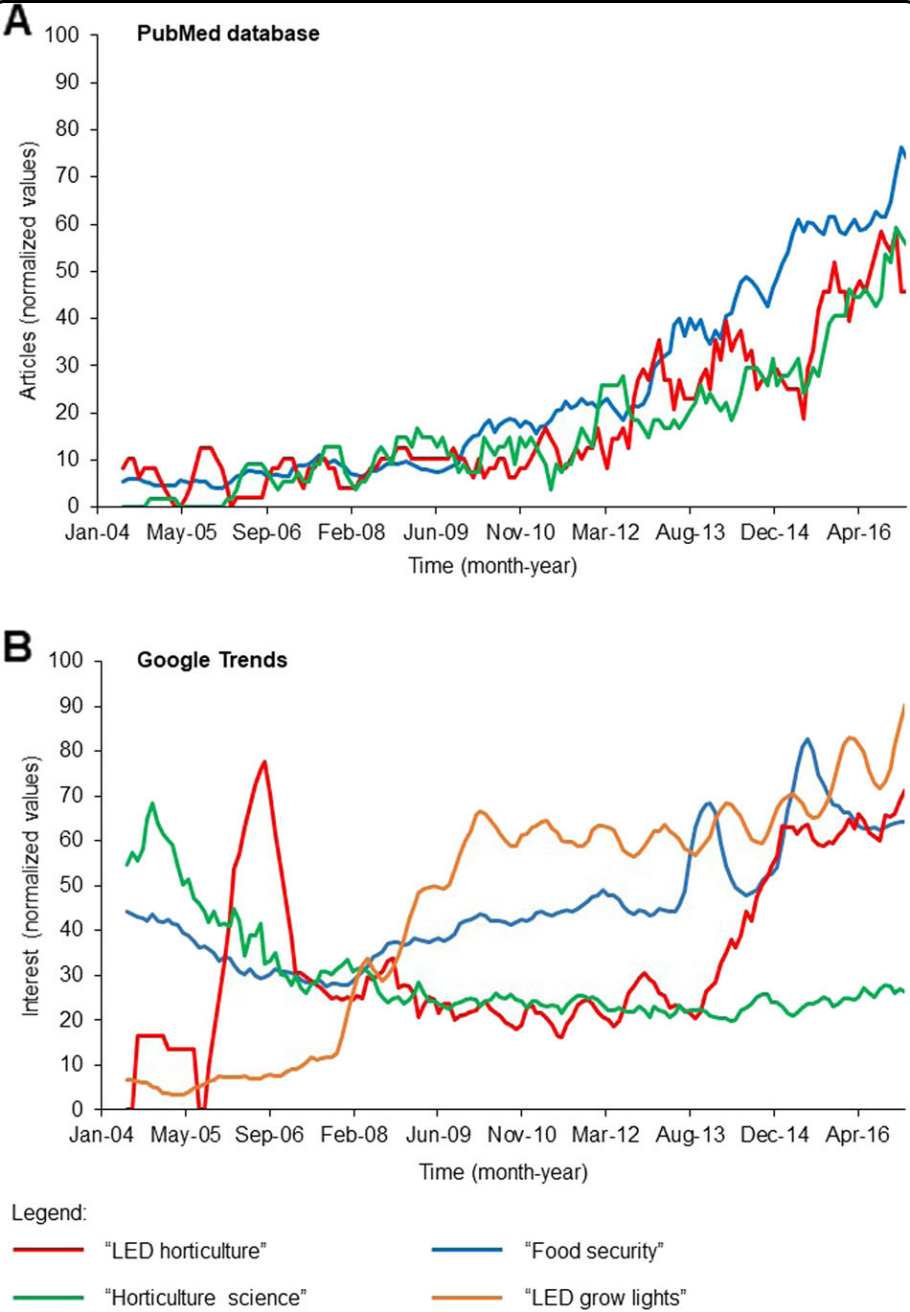
A recent survey of interest in indoor horticulture. The academic (**a**) and public (**b**) interests in ‘‘LED horticulture’’, ‘‘horticulture science’’, ‘‘food security’’, and ‘‘LED grow lights’’ within the period of January 2004 and February 2017. The respective search terms were used in the survey of public interest using Google Trends (https://trends.google.com/trends/) and to retrieve scholarly articles indexed in the PubMed database. The number of articles and Google Trend hits were normalized against the highest values of the respective search terms within the period between January 2004 and February 2017

In recent years, the use of electric lighting for horticulture has become an increasingly attractive option for plant cultivation either as supplementary or sole light source^9–14^. The application of light-emitting diodes (LEDs) lighting for plant growth was first documented in lettuce (*Lactuca sativa* L.), in which its development under a 16 h photoperiod of monochromatic red LED (660 nm peak) supplemented with a 30 μmol m^−2^ s^−1^ photosynthetic photon flux density (PPFD) of blue fluorescent lamps (400–500 nm range) at a total PPF of 325 ± 10 μmol m^−2^ s^−1^, was comparable to those grown under cool-white fluorescent and incandescent lamps^15^. Thereafter and concomitant with the advancement of LED technology, is a surge in the application of LED lighting in horticultural activities (Fig. 1). In general, public interests in this area as reflected by popular Google search terms ‘‘food security’’ and ‘‘horticulture science’’, has remained relatively consistent over the past decades, but interests in ‘‘LED horticulture’’ and ‘‘LED grow lights’’ have recorded marked increases in the last 5 years (Fig. 1b). These areas attracted comparable but increasing academic interests in the same period when searched against PubMed database using the same keywords (Fig. 1a). Notably, commercial ‘‘LED grow lights’’ were “in-trend” since 2008 presumably for small-scale ornamental plants in home- and office-based applications but the use of LED lighting technology for industrial-scale indoor horticulture (search term: ‘‘LED horticulture’’) only gained attention in the last 5 years as reflected by a sharp increase since August 2013 (Fig. 1b) thus implying a maturation of this technology for indoor horticulture applications. In recent years, indoor light sources have achieved significant progress in terms of energy- and cost-efficiencies. Concomitant with such technological advances is their application as electric lighting for indoor farming. In this regard, its application especially at an industrial scale, has been hampered at least in part by (1) the differential growth, morphology, and developmental behavior of plants cultivated under electric lightings, and (2) the lack of knowledge of how different light wavelengths influence plant performance in general and specific plant traits in particular. The former has been observed especially when using narrow waveband lights such as LEDs and lasers, which yield plants with variable traits and visual appearances^16–18^. Here, we pool together recent reports of electric lights for indoor farming and propose how a complementary phenotypic and functional genomics approach can be used to determine a set of light qualities and catalog a wealth of phenotype-specific molecular signatures that are tailored specially for indoor-grown plants.

## A recent account of indoor light qualities on plant growth and development

It is well-documented that light qualities (i.e., wavelengths and ratios) and their quantities can be used to manipulate plant characteristics as increasingly advanced lighting systems such as the narrow waveband LED lights and single-wavelength lasers have presented unparalleled technical and economical advantages^19–25^. Since the photoreceptors of plants absorb light corresponding to the regions of red and blue, a combination of appropriate ratios of LED lights are, therefore, required to achieve normal growth and photosynthetic capabilities such as that observed in a recent study on one Crassulacean acid metabolism (CAM) plant, the ice plant (*Mesembryanthemum crystallinum*) as well as in ornamental plants such as cabbage tree (*Cordyline australis*), weeping fig (*Ficus benjamina*), and gloxinia (*Sinningia speciose*)^26–28^. In the former for instance, red (670 nm peak) and blue (465 nm peak) LEDs provided at a ratio of 9:1 at a PPFD of 350 μmol m^−2^ s^−1^ and 16 h photoperiod, yielded the highest shoot and root biomass and shoot/root ratio in the succulent *M. crystallinum* plants compared to just red or blue LED lights alone^27^.

### Using electric lights to cultivate ornamental plants

Since it has been established that a combination of light wavelengths provided at suitable ratios can be used to cultivate plants in indoor environments, LED lights have been tested on many ornamental plants including but not limited to perilla, petunia, carnation, sunflower, rose, and chrysanthemum (Supplementary Table S1). Ornamental plants serve as good case studies for the testing of narrow-bandwidth electric lights such as LEDs, because they are in general smaller in size, consume fewer resources, have easily amenable features of broad esthetic values and are already accustomed to growth under indoor (home- or office-spaces) conditions compared to crop plants. In the case of Anthurium (*Anthurium andreanum*) and moth orchids (*Phalaenopsisis*), they not only displayed positive development but also yielded higher biochemical content when grown under LED lights (white light at 460 and 560 nm peaks; red at 660 nm peak and blue at 460 nm peak) compared to those grown under fluorescent lights (545–610 nm) (FL)^29^. In this study, the PPFD was maintained at 25 μmol m^−2^ s^−1^ under a 16 h photoperiod. Specifically, Anthurium showed the greatest plantlet length and number of leaves when treated with white, blue, and the combination of white, blue and red LEDs. In addition, more roots were observed in cultures treated with FLs and blue LEDs with 6.6 and 6.0 roots, respectively, than in cultures treated red LEDs (1.5 roots). Chlorophyll *a, b*, and total chlorophyll content were significantly higher in the blue LED treatment (0.692 mg g^−1^ fresh weight), while the lowest total chlorophyll content was found in the red LED and FL treatments yielding 0.327 and 0.375 mg g^−1^ fresh weights. Meanwhile, moth orchids displayed the greatest plantlet length and number of leaves when treated with FLs, white, and a combination of blue and red LEDs. Chlorophyll *a* content was significantly higher in the blue LED treatment (0.2813 mg g^−1^ fresh weight), while chlorophyll *b* content was higher in blue and the combination of blue and red LED treatments, yielding 0.1368 and 0.1468 mg g^−1^ fresh weights, respectively. Total chlorophyll (0.421875 mg g^−1^ fresh weight) was highest under blue LED while the lowest total chlorophyll content was found in FL treatments and white LEDs yielding 0.1810 and 0.2500 mg g^−1^ fresh weights, respectively^29^. We have summarized the effects of electric lights on the growth and development of other ornamentals studied in recent years in Supplementary Table S1. Based on the studies performed on different ornamentals, it is clear that narrow-bandwidth lights provided at suitable quality and quantity can induce esthetically valuable features.

### Using electric lights to cultivate crop plants

Unlike ornamentals, the use of electric lights on crop plants have a more specialized purpose, i.e., to improve the yield, biomass and/or nutrients for human consumption. Indoor horticulture has great potential to address food security since this form of crop farming is independent of geographical factors and weather conditions. It is, therefore, not surprising that narrow-bandwidth electric lights, in particular LEDs, have been broadly employed in studies involving crop plants especially green crops such as lettuce, cabbage, and artichoke (Supplementary Table S1). For instance, artichoke (*Cynara scolymus*) seedlings grown under red LED lights with a higher PPFDs than blue and white LEDs but only a third of natural light, yielded 60–100% more shoot dry weight and were 67–115% taller than those grown in greenhouse under natural light at a 16 h photoperiod. Nonetheless, seedlings grown under blue or white lights yielded 67–76% less biomass compared to greenhouse-grown seedlings^30^. In another report, cucumber (*Cucumis sativus*) plants grown under monochromatic LED lights (purple at 394.6 nm peak, blue at 452.5 nm peak, green at 522.5 nm peak, yellow at 594.5 nm peak and/or red at 628.6 nm peak at 350 μmol m^−2^ s^−1^ PPFD) for 12 h per day have reduced growth, CO_2_ assimilation rate, and quantum yield of photosystem II (PSII) electron transport compared to plants grown under white light control provided by natural light supplemented with incandescent reflector lamps^31^. On the contrary, red and blue LEDs improved the growth of lettuce (*Lactuca sativa*) in another study^32^. Another crop plant, Chinese cabbage (*Brassica campestris)* also showed improved vegetative and reproductive growth as determined by physical measurements and biochemical contents when treated with weak (80 μmol m^−2^ s^−1^ PPFD) blue, blue + red at 1:8 ratio and red LED lights in comparison to fluorescent lamps and sunlight at a 12 h photoperiod^33^. Conversely, an earlier study reported mostly lower growth rates for LED-illuminated cabbage^34^. While it is clear that electric lights have the potential to improve the yield and traits of crop plants in indoor farming, the effects are, however, inconsistent as they vary considerably across different studies. This inconsistency is further highlighted in Supplementary Table S1, which summarizes recent findings involving the use of electric lights on green crop plants.

### Using electric lights to cultivate fruits, herbs, and other economically valuable plants

Electric lights have also been employed on fruits such as tomato and strawberry as well as herbs such as mint, basil, and dill (Supplementary Table S1). For instance, electric lights can enhance fruit set and yield of strawberry plants although the fruit color appears less saturated as well as inducing greater biomass and phenolic content in basil (*Ocimum basilicum*)^16,18^. Different combinations of red and blue LED lights were able to increase oil yield in mints (*Mentha piperita, M. spicata and M. longifolia*), and improve growth and flower buds formation in basil (*Ocimum basilicum*)^35^ (see Supplementary Table S1 for details). Furthermore, electric lights have also been applied on plants that have other economic values such as medicinal plants and assisting reforestation goals. In the latter, narrow-bandwidth lights enable the selection of desirable traits of seedlings pre-cultivated in indoor environments prior to outdoor planting. In one particular example, the seedling quality traits of oak tree (*Quercus ithaburensis*) can be improved under blue, red, and far-red LED lights^36^ (see Supplementary Table S1 for details).

### Electric lights affect water usage efficiency of plants

One benefit of indoor horticulture is the reduction in water consumption. It was estimated that indoor plant cultivation can reduce water usage up to 90% compared to that used in open field farming. However, recent studies have suggested that the employment of electric lights negatively affected the water usage efficiency of crop and ornamental plants. For instance, the use of red and blue LED lights has resulted in reduced water usage efficiency in tomato (*Solanum lycopersicum*) and lisianthus (*Eustoma grandiflorum*) compared to those grown under high pressured sodium (HPS) lamps under a treatment of 100 ± 25 μmol m^−2^ s^−1^ photosynthetically active radiation (PAR) at a 16 h photoperiod. In both tomato and lisianthus, whole plant water usage efficiency decreased by 31% under the red and blue LED treatment compared to the HPS treatment. However, under red and white LED lights, whole plant water usage efficiency decreased by 25% for tomato and 15% for lisianthus in comparison to HPS treatment^37^. Therefore, in arid regions, it is necessary to consider the water usage efficiency of plants grown under electric lights in addition to trait improvements.

### Using electric lights to cultivate model plants and the relevance of such studies

Meanwhile, electric lights have also been tested on the model plant *Arabidopsis thaliana* albeit to a lesser extend compared to other economically valuable plants. While Arabidopsis can not represent crop plants and ornamentals, it however presents a fast and convenient way to study the effects of various parameters of electric lights including but not limited to wavelengths, ratios, and intensity. The knowledge gained from studies on Arabidopsis can in turn, serve as authoritative guides for the employment of electric lights on economically valuable plants. In one study, *Arabidopsis thaliana* plantlets grown under LED lights provided by two LED systems: type L18SP673 L, Valoya and the custom-made LED system (Roschwege) that combined LEDs with the light color warm white 3000K with 660 nm LEDs of moderate and 730 nm wavelength LEDs of low intensity, achieved higher rosette dry weight, seed mass, and developed faster compared to those grown under fluorescent lights (Osram L36W/830 und L36W/840, Osram) under a 180 μmol m^−2^ s^−1^ PPFD and at a 16 h photoperiod^38^. However, a recent study employing single-wavelength laser lights revealed a delay in the emergence of new leaves in Arabidopsis seedlings as well as their bolting and flowering times^17^. In this study, the single-wavelength laser beams were generated from a laser illumination system consisting of two diode-pumped solid-state lasers (Laserglow Technologies, Toronto, Canada), adjusted to a ratio of 9:1 of red (671 nm): blue (473 nm) and giving an average of total photon flux density of 90–100 μmol m^−2^ s^−1^. The same study also reported that leaves of laser-illuminated Arabidopsis plants have a lower total chlorophyll content and dry weight.

Currently, studies on *Arabidopsis thaliana* are limited because the outcome of such studies is often deemed not practical for ‘‘real-world’’ applications. There is currently a lack of genomics data to explain the inconsistencies and sometimes contradictory phenotypes in various crops grown under electric lights. This has hampered progress in the field of indoor farming especially in the transition from small-scale plant chambers or home- and office-spaces to large indoor plant factories. As such, a functional genomics approach that will be discussed in the following sections, can at least in part bridge this knowledge gap and explain these varying reports. However, in order to ascertain clear co-relations between the phenotypes and their corresponding genomic signatures in response to electric light qualities, which is the core message of this review, a well-characterized model plant such as Arabidopsis is required. Arabidopsis is by far the best characterized with regards to their genetics and is also easily amenable since there is already a large collection of light-related mutants. Cataloging functional genomics data that co-relates with the specific phenotypes of Arabidopsis can, therefore, form a basis that guides optimization works in other economically important crop and ornamental plants, hence the relevance of using Arabidopsis as a reference point.

### Mixed reports across model and representative crop plants require molecular validations

Taken together, different light qualities can result in varying degree of neutral, negative or positive effects on the growth and development of plants as characterized by their phenotypes, physiological properties, biochemical and biophysical contents, as well as altered responses to stresses. These effects were clearly demonstrated in a comparative study of LED lights (red at 660 nm peak and blue at 445 nm peak) and fluorescent light provided at a 14/10 h light/dark regimen with an intensity of 100–160 µmol s^−1^ across different widths and depths of the growth chamber, on the model plants *Arabidopsis thaliana* and *Nicotiana bentamiana*, and crop plants potato (*Solanum tuberosum*), oilseed rape (*Brassica napus*), and soybean (*Glycine max*)^39^. To further highlight the varying effects of electric light qualities on the phenotype, physical, and biochemical properties of plants, we have summarized as Supplementary Table S1, selected reports of economically important plant groups in the last 5 years (2013–2017). We group these findings in the following categories: ‘‘green crop plants’’, ‘‘fruits’’, ‘‘herbs’’, and ‘‘ornamental plants’’, where the mixed reports can be clearly seen. Although a broad coverage of model, crops, and ornamental plants have been examined under various electric lights, contradictory data have been reported across laboratories (see discussions in refs.^16,17^) and such inconsistencies can perhaps be reconciled through answers from a molecular perspective.

## Using functional genomics for economic gains

While various methods ranging from phenotypic, physical, and physiological measurements to biochemical and biophysical characterizations have been employed to study the effects of electric lights on the growth and development of plants, a molecular approach that employs a system-wide profiling of gene expression and function, is however under-utilized. Here, we explain citing recent examples, how functional genomics can be used to reveal crucial molecular signatures that can directly link the observed traits of plants grown to their light regimes. Functional genomics present a powerful molecular basis to gain insights on cellular biological processes and act as an inference point for biotechnological manipulations or indoor horticultural ambitions. Modern molecular tools in particular functional genomics, have generated a wealth of molecular data, which enabled mapping of previously elusive biochemical pathways governing plant responses to environmental cues including light perception and adaptation^40^. On the other hand, high throughput and increasingly sensitive instruments have contributed to the identification of missing components and assigning new functions to uncharacterized proteins^40^. Thus far, the major focal functional genomics tools that can be utilized to gain a systems-view on the influence of various light regimes on plant growth and development include transcriptomics, proteomics and metabolomics. Besides microarray technology, for transcriptome-wide studies, RNA-sequencing (RNA-seq) has also become a method of choice. RNA-seq technology provides a wide dynamic identification range of low-abundant transcripts and genetic variants thus permitting detection of more highly confident differentially expressed genes. Above all, functional genomics approach has been hastened by an increase in the number of available sequenced plant genomes. In the following sections, we discuss the employment of functional genomics in uncovering, at systems level, the physiological, biochemical and biophysical changes imposed by alterations in light regimes.

### Functional indicators diagnostic of light-induced regimes

Cellular processes predict the phenotype we observe and the efficiency of light absorption and utilization through the analysis of marker genes such as photosystem ii reaction center protein a (psbA), ascorbate peroxidase 1 (apx1), and light-harvesting chlorophyll *a*/*b*-binding protein 1 (LHCB1). This system-wide approach is diagnostic for functional and/or structural changes when induced by different light regimes. Distinct wavelengths of light impose different effects on plant physiology and morphology due to the differential sensitivity of photoreceptors. Various light responses are facilitated by a coordinated action of at least one photoreceptor^41^. Very few plant growth and development functional studies monitoring the biological role of different light routines and in particular LED lights, have been reported. Previously, microarray studies identified genes differentially regulated by green light and low red:far-red light ratio^42,43^. In the former study, etiolated Arabidopsis seedlings were treated with a short, single 100 µmol m^−2^ pulse of green light. The green light treatment induced expression of phytochrome A-regulated, nuclear encoded genes corroborating proper function of the sensitive phytochrome system. This is associated with a robust increase in stem elongation. On the contrary, in tobacco (*Nicotiana tabacum*), with the same temporal and fluence-response kinetics, plastid-encoded transcripts decreased in accumulation^42^. Taken together, the increase in stem growth rate and a decrease in plastid transcripts denotes a mechanism that influences progression of early commitment to light environment, assisting adaptation of seedling development during the critical process of early establishment^42^.

### Light regimes affect hormonal-related gene expression

It has been observed that by modifying low red:far-red ratio, light signaling and hormonal-related genes, particularly in the abscisic acid pathway were affected^43^. Using RNA-seq technology to analyze the molecular mechanisms by which various light qualities control Norway spruce seedling growth and phytohormone levels, a study showed that red light regulates the biosynthesis of gibberellic acid and thus, promotes stem elongation^44^. Furthermore, the authors show that blue light led to an increase in genes associated with secondary metabolites biosynthesis and potentially enhancing plant defenses^44^. Recently, RNA-seq technology was applied to study the effects of various wavebands of light on plant responses at transcriptional level^45^. In this study, grapes (*Vitis vinifera*) plantlets grown in vitro under white fluorescent lamp (FL40D-EX/38, Huadian CO., China), blue LEDs (peak at 440 nm), green LEDs (520 nm), and red LEDs (peak at 630 nm) lights exhibited over 600 differentially expressed genes with respect to white light. Here, taking advantage of the gene expression, phenotypic, and physiological data, the authors could link plant responses to different light spectrum and the expression patterns of particular sets of genes. For example, exposure to red and green light principally triggered responses associated with shade-avoidance syndrome (SAS) like enhanced stem elongation and decreased chlorophyll levels accompanied by the increased expression of genes encoding histones (H1, H2A, H2B, H3, H4), auxin-repressed protein, xyloglucan hydrolase, early light-induced protein (ELIP) and microtubule proteins^45,46^.

In addition, specific light treatments were observed to induce differential expression of many genes associated with diverse cellular functions, including those involved in ribosome pathway and primary metabolism such as starch and sucrose metabolic pathways, which is also supported by an increase of these metabolites in the plants. Notably, the authors highlighted a potential negative impact of adding sucrose to the culture media where it could at least in part, contribute to the observed increase in root growth and the upregulation of defense genes associated with SAS after exposure to red and green light. Unlike in the red and green light exposures, blue light-induced expression of genes associated with microtubules, chlorophyll biosynthesis, and sugar degradation. However, in the blue light accumulation of genes associated with auxin-repressed proteins and defense-related genes decreased. Generally, the observed effects from blue light exposure may explain the detected increase in leaf growth, chlorophyll synthesis, and chloroplast development as well as increase in chlorophyll *a*/chlorophyll *b* ratio in the leaves^45^. In this study, red light seems to promote high carbohydrate to protein ratio, whereas blue light has been observed to induce a low carbohydrate to protein ratio in plants^47^. Overall, plantlets grown under blue light have comparable growth to that observed under white light while exposure to red and green lights seem to cause shade stress on the plantlets^45^.

Light quality has also been shown to influence plant-mediated effects on herbivores and beneficial anthropods. Ultraviolet-B radiation (UV-B, 280–315 nm) treated plants attracted parasitoids. This can be attributed to UV-B light treatments promoting upregulation of oxylipin biosynthesis genes, which are also involved in jasmonic acid synthesis^48^. The increased transcription of these oxylipin biosynthesis genes and oxylipin biosynthesis could account for an increased emission of parasitoid-attracting volatiles from UV-B light treated plants. Previously, in sweet basil (*Ocimum basilicum*), an increase in the composition of volatile organic compounds was detected in essential oils following UV-B light treatment^49^. However, this observation could not be consistently reproduced in other plants suggesting that the indirect effects of UV light depletion for higher plants may be species specific^21^.

All in all, changes in light regimes or exposure to plants influence hormonal associated pathways, which impacts on gene or protein expression and the growth and development of the plants.

### Light regimes influence photosynthetic apparatus-associated genes

On another note, most genes related studies target either a single or a few genes that are linked to the photosynthetic apparatus or other light associated responses. For example, blue light with maximum intensity at 452.5 nm has been shown to induce expression of ten genes including fructose-1,6-bisphosphate, fructose-1,6-bisphosphate aldolase, ribulose-5-phosphate kinase, rubisco large subunit, rubisco small subunit, rubisco activase, triose-3-phosphate isomerase and ribulose phosphate epimerase all of which encode key enzymes in the Calvin cycle^32^. In addition, blue light acts as a catalytic wavelength in acquiring high quantum yields of photosynthesis and activating respiration^21^. On the contrary, green (with maximum intensity at 522.5 nm), yellow (594.5 nm), and red (628.6 nm) lights cause downregulation of these key Calvin cycle enzymes^32^.

Another study examining the impact of single-wavelength laser light (adjusted to a ratio of 9:1 of red (671 nm) : blue (473 nm) giving an average total photon flux density of 90–100 µmol m^−2^ s^−1^) on plant growth and development looked at the expression levels of six photosynthetic marker genes, each representing a main component of the photosynthetic system^17^. Four of the six genes (photosystem I p700 chlorophyll *a* apoprotein A1, ferredoxin 2, psbA, and LHCB1) showed lower expression in laser-grown plants than cool-white fluorescent grown plants. The low expression of *psbA*, a gene that is generally associated with photo-inhibition and the photo-damaged of PSII particularly when the light absorption exceeds consumption^50–52^, suggests that the laser-grown plants had reduced photo-inhibition. In addition, the expression of two light-stress marker genes, ascorbate peroxidase 1 and glutathione s-transferase was observed to decrease in plants grown under laser light compared to white light signifying that the laser illumination conditions induce less stress than the white fluorescent light (for review see ref.^17^).

### Influence of light regimes on the protein abundance

Just like the transcriptional gene regulation studies, proteomics studies reflecting the fate of translational changes influenced by different light regimes are yet to be fully explored. Similar to influencing transcriptional changes, different light wavelengths induce changes in the global proteome of cells. Strong blue light has been shown to activate the incorporation of carbon in amino acids thereby inhibiting the biosynthesis of starch in leaf chloroplasts while increasing the biosynthesis of proteins^47^. However, this increase of protein biosynthesis induced by exposure to blue light can be abolished by prolonged exposure of plants to red light^53^. Recently, a global proteomics comparative study was performed on laser- and white light-grown plants to evaluate the impact of variable light exposure on the proteome of the plants as well as relate this molecular data to the physiological data collected from the same plants^17^. In this study, plants grown under laser illumination have lower expression of proteins indicative of light and radiation stress responses. There were 115 differentially regulated proteins of which only 17 proteins were upregulated. The majority, 98 proteins, were downregulated and of these 43 were annotated as localized in the chloroplast and 12 of the 43 proteins are involved in photosynthesis. Of important to note is that seven light-harvesting chlorophyll-protein (LHC) complexes including LHCB1.4 (At2g34430) and LHCB3 (At5g54270) were among the most downregulated proteins in the laser-illuminated plants. This corroborates well with the observed changes at the transcripts level. The LHC proteins play an important role in fine-tuning the amount of light energy to be channeled to the reaction centers, a process that enables plants to adapt to a wide spectrum of light environments to drive photosynthesis^54^. Since the LHC family of proteins is light-stress induced, a reduction in its abundance is indicative of reduced photo-oxidative stress under laser-illuminated plants. Furthermore, 16 proteins enriched in gene ontology category “response to light stress” were downregulated in the laser-illuminated plants, lending more support to the concept that laser-light regime induces less stress on plants than white fluorescent light. Phenotypic and biochemical data characterization further supported these observations at molecular levels, including gene expression patterns of marker genes. For example, phenotypically, the average leaf length and area of the first two leaf pairs were observed to be higher in laser-illuminated plants than the white fluorescent light (control) exposed plants but bolting and flowering times were slightly delayed. The authors argued that the delay in flowering time was most likely due to the absence of far-red light that has been shown to promote flowering^55,56^. From a biochemical point of view, laser-grown plants leaves had lower total chlorophyll content compared to control plants. Similarly, it has previously been reported that chlorophyll levels in spinach (*Spinacea oleracea*) are reduced under a 9:1 ratio of red LED (660 nm) and blue fluorescent lamp at a total PPFD of 282 µmol m^−2^ s^−1^ as compared to cool-white fluorescent light^57^. Additionally, absence of elevation of light-stress response proteins in the proteomics data is indicative that the laser-light regime, which was employed is suitable and sufficient for plant growth^17^.

## A highly tailored approach to indoor plant cultivation

Single-wavelength lights (e.g., lasers) and their tunability can conceivably provide a highly tailored approach to indoor plant cultivation, allowing for more flexibility than narrow waveband LEDs^58–60^. A recent report on the model plant *Arabidopsis thaliana*, showed that plants can complete a full cycle of growth and development under single-wavelength lights comprising of adjusted red (671 nm) : blue (473 nm) ratio of 9:1^17^. This has paved the way for more comprehensive light formulae containing well-defined mixtures of light wavelengths, intensities and ratios, to tailor for the cultivation of different plants and to obtain specific plant traits for indoor horticulture activities^59^.

**Fig. 2:**
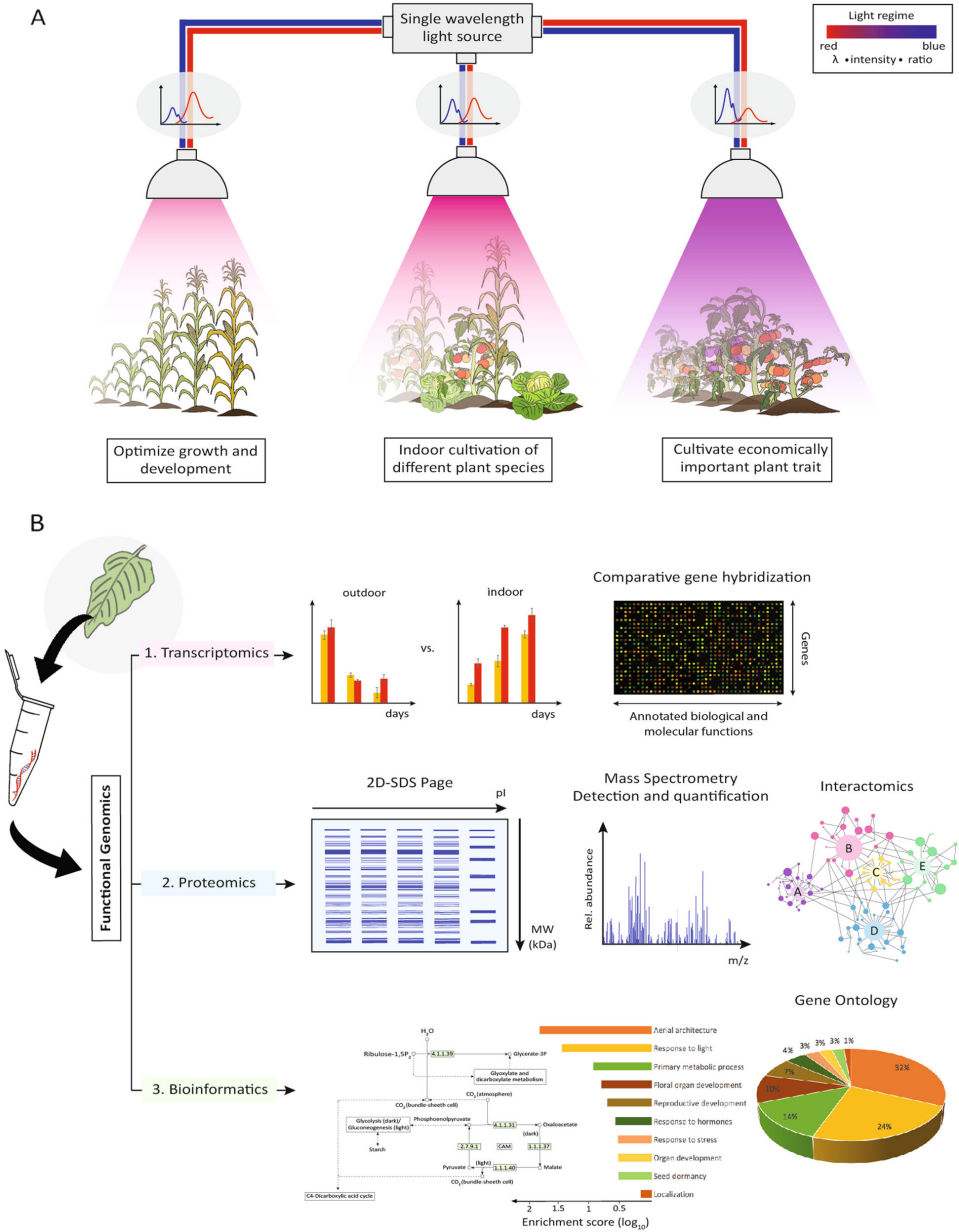
An illustration of a highly tailored indoor horticulture approach. **a** Light regimes comprising of well-defined mixtures of single-wavelength lights in their pre-determined optimal ratios and intensities can be used to grow different species of plants, cultivate economically important plant traits, and optimize the growth and development stages of plants in highly controlled indoor environments. **b** Plants displaying favorable traits and growth parameters under the optimized light regimes are subjected to functional genomics where their underlying molecular signatures can be harvested for biotechnological innovations to produce plant traits and yield that are economically attractive especially when grown under light regimes of indoor environments

We, therefore, propose the use of a complementary phenotypic and functional genomics approach to determine the optimal light conditions for plant growth in indoor environments using *Arabidopsis thaliana* as the model plant since the Arabidopsis plant has a short life span, a large collection of light-related mutants and a fully sequenced genome and proteome, and importantly, the findings can be easily translated to crop plants and where necessary further optimize light conditions with model crop plants like rice (*Oryza sativa*) or tomato^61^. As illustrated in Fig. 2, we propose that a preliminary broad screening that document extensively the phenotypes of model plants grown under different light regimes (i.e., different wavelength combinations, intensities and ratios) should be conducted using narrow waveband and/or single-wavelength lights to determine the optimal light conditions that yield economically important traits. Subsequently, a functional genomics approach involving transcriptomics and proteomic analyses of plants displaying economically beneficial traits under the respective light regimes can be conducted, to ascertain the molecular signatures governing the regulation of genes involved in expression of these desirable phenotypes^62^. A catalog of phenotype-specific molecular signatures can act as an authoritative guide to determine the optimal light qualities for crop plants, for cultivating different traits and for potential biotechnological innovations that are specific for indoor horticulture applications^40,63^. Given that the knowledge gained from a complementary phenotypic and functional genomics study can be harvested for economical gains, this highly tailored approach to indoor horticulture can, therefore, contribute to sustainable food production.

## Electronic supplementary material

Supplementary Table S1
