# Supplementary material for: Towards a tailored indoor horticulture: a functional genomics guided phenotypic approach
Source: Hortic Res. 2018 Nov 1;5:68. doi: 10.1038/s41438-018-0065-7 (PMC6210194; doi:10.1038/s41438-018-0065-7)
Supplement: Supplementary file 1 — Supplementary Table S1 [file 41438_2018_65_MOESM1_ESM.docx]

**Supplemental Information for:**

**Title**

Towards a tailored indoor horticulture: A functional genomics guided phenotypic approach

**Authors**

Claudius Marondedze^1^, Xinyun Liu^2^, Shihui Huang^2^, Cynthia Wong^3^, Xuan Zhou^2^, Xutong Pan^2^, Xuechen Tian^2^, Nuo Xu^2^, Aloysius Wong^2^*

**Affiliations**

^1^ Laboratoire de Physiologie Cellulaire et Végétale, Université Grenoble Alpes, CEA/DRF/BIG, INRA UMR1417, CNRS UMR5168, 38054 Grenoble Cedex 9, France.

^2^ Department of Biology, Wenzhou-Kean University, 88 Daxue Road, Ouhai, Wenzhou, Zhejiang Province, 325060, China.

^3^ Department of Cell and Systems Biology, University of Toronto, 25 Willcocks Street, Toronto, ON, M5S 3B2, Canada.

* Corresponding author at:

Wenzhou-Kean University, Department of Biology

88 Daxue Road, Ouhai, Wenzhou, Zhejiang Province, 325060, China.

Tel: +86-138-5771-3894 E-mail address: [alwong@kean.edu](mailto:alwong@kean.edu) (A. Wong)

**This supplementary information consists of one supplementary table (Supplementary Table S1).**

**Supplementary Table S1** Effect of electric lightings on plant growth and development. Selected reports on green crop plants, fruits, ornamentals, herbs, medicinal plants and *Arabidopsis thaliana* from the last 5 years (from 2013) are represented in this table.

| Plant | Light source, quality and regime | Phenotypic, physical and biochemical properties | Ref. |
| --- | --- | --- | --- |
| *^a^* Lettuce  *Lactuca sativa L.* cv. ‘Grizzly’ | LED (100% red, 100% blue, 70% red + 30% blue, and 100% white) vs. natural light in greenhouse with a PPFD of 300 µmol m^−2^ s^−1^ at 14 h photoperiod. | Fresh mass of plants grown under 100% blue and 70% red + 30% blue LEDs, chlorophyll and carotenoid concentrations of plants grown under 70% red + 30% blue LEDs and vitamin C content of plants grown under 100% blue LEDs are higher compared to those grown under natural light in greenhouse. | 1{Amoozgar, 2017 #158;Amoozgar, 2017 #158} |
| *^a^* Green Oak Leaf lettuce  *Lactuca sativa var. crispa* ‘Green Oak Leaf’ | Four alternating light treatments (R/B) had the same 8.64 μmol m^−2^ daily light integral (DLI) and similar R:B ratio (2:1), but different R/B alternating intervals that were respectively 8 h, 4 h, 2 h, and 1 h during a 16 h photoperiod. Two simultaneous light treatments, one of which (RB) had the same DLI and energy consumption with the alternating light treatments, while the other (RB’) had the same photoperiod with the alternating light treatments were set up to compare the concurrently and alternately provided R and B. | Plants with simultaneous RB appeared the sparsest while those with RB’ looked the most compact. Plant height/width and leaf length/width were all the highest under R/B (8 h) followed by R/B (1 h). Lettuce biomass under RB’ was significantly higher than others, more than twice that under RB, R/B (4 h) and R/B (2 h), but less than twice that under R/B (8 h) and R/B (1 h). RB’ significantly decreased the soluble sugar content by 9%–32% while increased crude fiber content by 14%–39% compared with others. Significantly higher ascorbic acid content as well as lower nitrate content were detected in lettuce under R/B (4 h) and R/B (2 h), while significantly lower ascorbic acid content as well as higher nitrate content were detected in lettuce under R/B (8 h) and R/B (1 h). In all, based on the same energy consumption, R/B (8 h) and R/B (1 h) resulted in higher yield, while R/B (4 h) and R/B (2 h) brought about higher nutritive value compared with the concurrent light RB. | 2 |
| *^a^* Lamb’s lettuce *Valerianella locusta* L. | Four different LED spectra used to extend the photoperiod (16 h day/8 h night) were tested: 10R:0B (100% red), 9R:1B (90% red, 10% blue), 8R:2B (80% red, 20% blue), and 7R:3B (70% red, 30% blue). The control plants were illuminated by HPS lamps. All treatments were at approximately 200 μmol m^-2^ s^-1^. | 10R:0B LED light treatment resulted in the highest yield, but low concentrations of ascorbic acid, xantophylls, and phenolic compounds. Supplemental lighting with HPS lamps resulted in high concentrations of nitrate and carotenoids, but low fresh mass, ascorbic acid, and total phenols content. The addition of blue light in the spectrum increased antioxidant properties. High yields with the most satisfactory composition of soluble sugars, ascorbic acid, carotenoids, and polyphenols, as well as high antioxidant properties and reduced nitrate levels, in the leaves of the tested cultivars were obtained with 8R:2B LED lighting. | 3 |
| *^a^* Lettuce Leaf and Red Rubin-mountain Athos hybrid cultivars  *^c^* Basil  *Ocimum basilicum* | Four LED light treatments: AP673L (high red and high red:far-red), G2 (high red and low red:far-red), AP67 (moderate blue and redand low red:far-red), and NS1 (high blue and green, high red:far-red and 1% ultraviolet)] with different colors mixing UV, blue, green, red and far-red, vs. fluorescent light controls (FL, high blue, green and red:far-red) PPFD of 200 ± 20 µmol m^−2^ s^−1^ at 14 h photoperiod. | G2, control and AP67 treatments for Lettuce Leaf, and G2 for Red Rubin hybrid had higher growth rate. Roots of Lettuce Leaf were significantly longer under AP673L compared to NS1, while Red Rubin hybrid showed no significant differences. Total biomass was significantly greater under NS1, AP67 and G2 compared to the Control, for both cultivars. For both Lettuce Leaf and Red Rubin hybrid, root:shoot ratio (R/S) was favored under NS1, whereas the control had the lowest impact. Leaf area of both cultivars was greater under the control. Total phenolic content of Lettuce Leaf was significantly higher under NS1 compared to the rest of the treatments, while in Red Rubin hybrid, NS1 had significantly higher total phenolic content compared to the control and G2. | 4 |
| *^a^* Green Oak Leaf lettuce  *Lactuca sativa var. crispa* ‘Green Oak Leaf’ | Mixed light qualities of white light LED and a different supplemental light such as blue (B), green (G), yellow (Y), red (R), and far-red (Fr) LEDs. White LED (W) was used as control with a PPFD value of approximately 135 µmol m^−2^ s^−1^ and the basal white light in each treatment was 105 µmol m^−2^ s^−1^ supplemented by approximately 30 µmol m^−2^ s^−1^ colored LED. | Supplemental lights led to obvious morphological changes where plants with WR appeared compact and vigorous while those with WY and WFr looked sparse and twisted. Dwarfed plants with large leaves were detected under WB. Compared with those grown in the white light control, the fresh weight of shoots increased by 63.2% and 21.7% with supplemental R and B respectively, while decreased by 35.9% with supplemental Fr. Chlorophyll and carotenoid contents were significantly higher with supplemental R and B than other treatments. Supplemental B and G resulted in decrease of nitrate content. Supplemental G significantly promoted soluble sugar accumulation. Supplemental Fr increased S/R ratio and ascorbic acid accumulation but resulted in lower biomass and pigment contents. | 5 |
| *^a^* Lettuce  *Lactuca sativa* L. var. green-skirt | Plants grown under different combinations of red, blue (0, 10, 20, and 30%), and green (0 and 10%) light at 150 ± 15 μmol m^-2^ s^-1^ of PPFD at 16 h photoperiod with white fluorescent lamp as control. | The leaf photosynthetic rate was highest under 80% red and 20% blue light and decreased significantly with the addition of green light and the absence of blue light. As the fraction of blue light increased, leaf size and plant growth decreased significantly. However, while the addition of green light considerably reduced the leaf photosynthetic rate, it did not reduce plant growth. the addition of 10% (15 μmol m^-2^ s^-1^) green light did not have a positive effect on the growth of lettuce. | 6 |
| *^a^* Red leaf lettuce *Lactuca sativa L. ‘Sunmang’* | In study I, six LED sources were used to determine the effects of supplementary white LEDs (RGB 7:1:1, 7:1:2, RWB 7:1:2, 7:2:1, 8:1:1, 8:2:0 on lettuce) with fluorescent lamps as control. In study II, pulsed RWB 7:2:1 LED treatments (30, 10, 1 kHz with a 50 or 75% duty ratio) were applied. PPFD of 173 ± 5 μmol m^−2^ s^−1^ for 12 h photoperiod. | In study I, the application of red and blue fractions improved plant growth characteristics and the accumulation of antioxidant phenolic compounds, respectively. In addition, the application of green light increased plant growth, including the fresh and dry weights of shoots and roots, as well as leaf area. However, the substitution of green LEDs with white LEDs induced approximately 3.4-times higher light and energy use efficiency. In study II, the growth characteristics and photosynthesis of lettuce were affected by various combinations of duty ratio and frequency. In particular, biomass under a 1 kHz 75% duty ratio of pulsed LEDs was not significantly different from that of the control (continuous LEDs). Moreover, no significant difference in leaf photosynthetic rate was observed between any pulsed LED treatment utilizing a 75% duty ratio versus continuous LEDs. However, some pulsed LED treatments may potentially improve light and energy use efficiency compared to continuous LEDs. | 7 |
| *^a^* Cos lettuce *Lactuca sativa* L. | Plants treated with blue (463 nm peak) and red (656 nm peak) LED lights at a PPFD of 90 μmol m^-2^ s^-1^ for 14 h per day. The red LED light irradiation starting time was simultaneous to or delayed 1, 4, or 7 h from the blue LED light irradiation starting time. | Shoot fresh weight of plants grown under irradiation patterns with red LED light irradiation being delayed 4 or 7 h from blue LED light irradiation was significantly greater than that under irradiation patterns with blue and red LED light irradiation starting simultaneously. The growth of plants can be promoted merely by tempo-rally shifting the irradiation hours of blue and red LED lights. The diurnal PPFD change contributed to the growth-promoting effect and that blue and/or red light monochromatic irradiation increased the total leaf area of plants. | 8 |
| *^a^* Red leaf lettuce  *Lactuca sativa* ‘Sunmang’ | Plants are treated with various ratios of red (660 nm peak) (R) and far-red (732 nm peak) (FR) LEDs (R/FR = 0.7, 1.2, 4.1, and 8.6), only red LEDs (RED), or fluorescent lamps (control) at a PPF of 130 ± 5 µmol m^−2^ s^−1^ and 12 h photoperiod | Fresh and dry weights and leaf area in all R/FR treatments were higher than those in the control at 22 days of treatment. The R/FR 1.2 had the highest values among R/FR treatments. The number of leaves appeared to increase as R/FR ratio increased. The specific leaf weights in the R/FR ratio of 0.7, 1.2, and 8.6 were similar to the control at 22 days of treatment. The SPAD values in all R/FR treatments were lower than that in the control. All R/FR treatments led to a longer leaf shape than the control. The percentage of cells in the G2M phase, indicating the cell division rate, increased in the R/FR treatments after 4 days of treatment, which supported the growth improvement in the R/FR treatments. The Fv/Fm and the photosynthetic rate in all treatments decreased due to the absence of blue light. | 9 |
| *^a^* Lettuce  Red leaf *Lactuca sativa L. ‘Sunmang’* and green leaf *Lactuca sativa L.* ‘Grand Rapid TBR’. | Six LED lighting sources (R:B = 9:1, 8:2, 7:3; R:G:B = 9:1:0, 8:1:1, 7:1:2) were manufactured to emit red (655 nm), blue (456 nm), or green (518 nm) lights under PPFD of 173 ± 3 μmol m^−2^ s^−1^ for 12 h photoperiod. | Red LEDs were found to improve growth characteristics such as fresh and dry weights of shoots and roots, and leaf area in combination with blue LEDs. The substitution of blue with green LEDs in the presence of a fixed proportion of red LEDs enhanced the growth of lettuce. | 10 |
| *^a^* Lamb’s lettuce  *Valerianella locusta* | LED and HPS lights (PPFD of 200 µmol m^−2^ s^−1^ at a 16 h photoperiod) as a supplemental to solar radiation in winter growing. Plants grown under sole red (660 nm), combination of red (660 nm) and blue (430 nm) in different ratios (90R/10B, 70R/30B and 50R/50B), warm white LEDs and HPS lamps. | Fresh weight of rosettes, dry matter, soluble sugars, total phenols content and radical scavenging activity were the greatest under 90R/10B LED lamps. The least efficient in the enhancing of lamb’s lettuce yield proved to be the supplemental lighting with white LED lamps. The lowest level of ascorbic acid content was shown in the case of the plants illuminated with HPS and 100% red light. | 11 |
| *^a^* Leaf lettuce *Lactuca sativa var. crispa* | LEDs emitting different bands of short wavelengths red (R) 623–673nm, light red (Lr) 599–644nm, blue (B) 427–478nm, light-blue (Lb) 435–489nm, cyan (C) 466–532nm, green (G) 494–564nm, and ultraviolet-A (UV-A) 383–426nm at PPFD of 300 ± 12 µmol m^−2^ s^−1^ at 18 h photoperiod were used in the following ratios: LrLb (61.5% and 38.5%, respectively), RCB (42.8%, 30.0%, and 27.2%, respectively), LrLbG (49.0%, 36.1%, and 14.9%) and RBUV-A (52.9%, 37.0%, and 10.1%). | RBUV-A and RCB LED irradiations during the vegetative stage could increase the shoot fresh mass of leaf lettuce. In addition, providing different LED light formulas to lettuce plants at the seedling and vegetative stages resulted in tremendous differences in nitrate contents, especially with the use of two light formulas with and without reversal of lighting sequence. Adoption of LrLbG or LrLb LED irradiation during different growth stages resulted in the lowest physiological indices of lettuce plants. | 12 |
| *^a^* Red pak choi *Brassica rapa var. chinensis*,  *^a^* Mustard *Brassica juncea L.*, and  *^a^* Tatsoi  *Brassica rapa var. rosularis* | Pulsed LED lighting versus continuous lighting. Plant treated with HPS lamps supplemented with monochromatic (455, 470, 505, 590, and 627 nm) LEDs [total PPFD of 200 ± 10 μmol m^−2^ s^−1^ at 16 h photoperiod]. For pulsed light treatments, the frequencies at 2, 32, 256, and 1024 Hz with a duty cycle of 50% monochromatic LEDs were applied. The results were compared to those under the continuous light (0 Hz) condition in terms of total phenolic content, anthocyanins, and antiradical activity (DPPH). | The most positive effects of 2, 256, and 1024 Hz for total phenolic compounds in mustard under all wavelength LEDs were achieved. The LED frequencies at 2 and 32 Hz were the most suitable for accumulation of anthocyanins in red pak choi and tatsoi. The highest antiradical activity under the treatments of 32, 256, and 1024 Hz in mustard and under the 2 Hz frequency in red pak choi and tatsoi was determined. | 13 |
| *^a^* Artichoke  *Cynara cardunculus* var. “Green Globe,” “Cardoon,” and  “Violetto” | Seedlings were treated with natural light, red, blue and white LEDs at varying PPFDs with spectral quality assessment showed that PPFD was highest under  red LED light, but only a third of the total PPFD under natural light. Light treatments were provided at 16 h photoperiod. | Seedlings grown under red light showed 60 – 100% more shoot dry weight and were 67 – 115% taller than seedlings grown in the greenhouse. However, seedlings under blue or white light conditions showed 67 – 76% less in biomass compared to greenhouse-grown seedlings. Overall, plant response of seedlings under red light condition was much better compared to greenhouse-grown seedlings. | 14 |
| *^a^* Chinese cabbage *Brassica сhinensis* L. | Plants treated with varying proportion of red to white LED light ratios, photon flux density and intermittent light cycles. | The light regime: PPFD of 500 μmol m^−2^ s^−1^, about 70%-proportion of the red component of the light spectrum (PPFD _LED red_ /PPFD _LED white_ = 1.5) and the duty cycle with a period of 501 μs, is optimal for crop productivity measured in terms of dry weight. | 15 |
| *^a^* Mustard  *Brassica*  *juncea* L. ‘Red Lion’  *^a^* Red pak choi *Brassica rapa var. chinensis* ‘Rubi F1’ and  *^a^* Tatsoi  *Brassica rapa var. rosularis* | Two experiments were performed: (1) evaluation of LED irradiance levels at PPFD 545, 440, 330, 220, and 110 µmol m^−2^ s^−1^ using a standard set of LED lighting system consisting of 447-, 638-, 665-, and 731 nm LEDs and (2) evaluation of the effects of 520-, 595-, and 622 nm LEDs supplemental to the standard set of LEDs. All experiments performed at 16 h photoperiod. | Concentrations of various carotenoids in Red pak choi and Tatsoi were higher under illumination of 330 – 440 µmol m^−2^ s^−1^ and at 110 – 220 µmol m^−2^ s^−1^ in Mustard. All supplemental wavelengths increased total carotenoid content in Mustard but decreased it in Red pak choi. Carotenoid content increased in tatsoi under supplemental yellow light. | 16 |
| *^a^* Rapeseed  *Brassica napus* L. | Plants grown under 60 µmol m^−2^ s^−1^ PPF for a 12 h photoperiod under the following six different light qualities: fluorescent lamps (FL), blue (440 nm peak) LEDs (B), red (660 nm peak) LED (R), and three mixtures of B plus R (3:1, 1:1, 1:3) LED. | The proliferation rate was greater in plantlets that were cultured under B light than those under FL. The differentiation rate, fresh mass, dry mass, concentration of chlorophyll a, soluble sugar concentration, stem diameter, leaf stomata abaxial surface length, adaxial surface stomata frequency and transplantation survival rate were greater in plantlets that were cultured under B:R = 3:1 light than under FL. The concentration of starch and the spongy tissue length were higher in plantlets cultured under R light than those under FL. | 17 |
| *^a^* Stevia  *Stevia rebaudiana Bertoni* | Three different light wavelengths using LEDs: red (R), blue (B), and a combination of red and white (R + W) light (mixed at a 1:1 energy ratio) were tested on plants with white fluorescent light (WF) and darkness (D) used as control treatments (respectively, control 1 and control 2). Continuous light cycle at PPFD 60 μmol m^−2^ s^−1^ was applied. | Blue LED light increased seed germination and affected the development of the largest number of leaves and roots in 4-week-old Stevia plantlets. Red LED light however, significantly increased the length of stems and roots, although there was not correlation with the fresh weight (FW). Blue LED light also positively affected the carotenoids concentration, whilst the highest concentration of chlorophyll a and b was found, in plantlets grown under white fluorescent light. The less favourable effect on the synthesis of pigments was exerted by red LED light. | 18 |
| *^b^* Tomato  *Solanum lycopersicum* cv. “Bonny Best” and  *^d^* *Eustoma grandiflorum* cv. “Flare” | Plants treated ambient (natural light/control), and three supplemental light treatments: HPS, red-blue (RB) LEDs and red-white (RW) LEDs, each providing 100 ± 25 μmol m^−2^ s^−1^ of PAR at a 16 h photoperiod. | A decrease in whole plant water usage efficiency (WUE) was seen in both crops under both RB and RW LEDs when compared to HPS light. Whole plant WUE was decreased by 31% under the RB LED treatment for both crops compared to the HPS treatment. Tomato whole plant WUE was decreased by 25% and lisianthus whole plant WUE was decreased by 15% when compared to the HPS treatment when grown under RW LED. | 19 |
| *^b^* Tomato  *Solanum lycopersicum* L. | LED (33% red + 33% green + 33% blue and 66% red + 33% blue) vs. white fluorescent lamps with a PPFD of 200 µmol m^−2^ s^−1^ at 12 h photoperiod. | LED grown plants have lower height, biomass and leaf lamina thickness and area but higher CO_2_ assimilation rate and bigger stomata size. LED grown plants produced lower β-phellandrene but higher α-pinene, carene and α-terpinene. | 20 |
| *^b^* Tomato  *Solanum*  *lycopersicum* ‘Espero’ | HPS or LED light (blue; peaks at 402, 419, and 445 nm, and red/far-red; peaks at 663 and 737 nm) and one treatment with alternating HPS and LED light (three days each) with a PPFD of 100 – 120 µmol m^−2^ s^−1^ at 16 h photoperiod. | Plants grown in alternating LED and HPS lamps had lower fresh weight as compared with HPS. Plants subjected to alternating light regimes generally resembled LED treatment plants more than HPS plants. Leaf transmittance and reflectance were higher for leaves grown in HPS light. | 21 |
| *^b^* Tomato *Solanum lycopersicum* | Seedlings are irradiated daily for 12 h with white LED light at 300 200 µmol m^−2^ s^−1^ PPFD. During each 12 h nighttime interval, all plants except for the controls were irradiated with white LED light at 150 or 300 µmol m^−2^ s^−1^, or alternatively with blue, orange, or red LED light at 150 µmol m^−2^ s^−1^. | Plants irradiated overnight exhibited injuries, with their visually rated severity of injury increased as the PPFD of nighttime white light was increased. In plants irradiated overnight at 150 µmol m^−2^ s^−1^, a higher degree of injury was experienced under blue light than under orange and red light. Overnight white-light irradiation at 150 µmol m^−2^ s^−1^ PPFD promoted dry matter production compared with the no-irradiation control, but no further increase was observed at a PPFD of 300 µmol m^−2^ s^−1^. Among different spectra, blue light was less effective than orange light at promoting dry matter production when white light was supplied during the day. | 22 |
| *^b^* Tomato seedlings  *Lycopersicon esculentum* L., cv. Superdoterang | Plant are treated with continuous illumination of 376 nm UV-A LED, 658 nm red LEDs or the red LED supplemented with two irradiation levels of the UV-A. All plants received PPFD of 90 µmol m^−2^ s^−1^. | The growth and development of tomato seedlings were significantly enhanced under the red light supplemented with the UV-A. Under the UV-A treatments, the tomato seedlings became more compact, the growth of plant organs was balanced, the leaf area was increased, and the total plant fresh and dry weights were also enhanced. 376 nm UV-A from LEDs had a beneficial effect on the growth and development of tomato seedlings as similarly to the blue light. | 23 |
| *^b^* Strawberry  *Fragaria × ananassa Duch. ‘HS138’* and  *Fragaria vesca* | Plants grown under three types of blue and red LEDs (blue light peak wavelength: 405, 450, and 470 nm; red light peak wavelength: 630, 660, and 685 nm) were provided continuously at PPF of 80 µmol m^−2^ s^−1^. | All blue light from the various peak LED types promoted more flowering compared with red light (630 and 660 nm except for 685 nm). The longer wavelength among the red light range positively correlated with earlier flowering, whereas the number of days to anthesis did not significantly differ among blue LED treatment wavelengths, irrespective of peak wavelength. | 24 |
| *^b^* Strawberry  *Fragaria ×*  *ananassa Duch.* cv. Daewang | A growth chamber (GC) illuminated with LED lights as the sole light source and a plastic greenhouse (PG) which was given supplemental LED light (blue, red and blue plus red) in addition to ambient light. | Plants cultivated in the PG with supplementary LED lights yielded much higher production of fruits than those cultivated in the GC. Fruits harvested in the PG contain higher levels of organic acids than those harvested in the G. A remarkably higher production of fruits was achieved in the PG when ambient light was supplemented with either blue LED light or combined blue and red LED light. Greater accumulation of organic acids and phytochemicals such as phenolic compounds were observed in the fruits that had been cultivated in the PG when ambient light was supplemented with either red LED light or combined blue and red LED lights. | 25 |
| *^b^* Strawberry  cv. ‘Camarosa’ | Plantlets treated with LEDs at 90% red (660 nm peak) and 10% blue (460 nm peak), 70% red and30% blue, 50% red and 50% blue, 30% red and 70% blue wavelengths, and fluorescent lamps (control) at 50 μmol m^-2^ s^-1^ PPFD for 16 h per day. | The 90% red and 10% blue LED was optimal for in vitro development. The 70% red and 30% blue LED was effective for both in vitro and ex vitro growth. In comparison with the control, all LEDs significantly promoted plantlet development and increased survival rate, shoot and root biomass, root number and length, leaf number and area, and chlorophyll content. | 26 |
| *^c^* Basil  *Ocimum basilicum* L. | Plants grown under four different supplemental LED light treatments; 80 % Red/20 % Blue, 80 % Red/20 % Blue + UV-A, 40 % Red/60 % Blue and 80 % Red/20 % Green provided at 150 μmol m^-2^ s^-1^ of PPFD in addition to the natural light in greenhouse at 20 h photoperiod. | Increasing ratios of blue light having negative effects, and green light having positive effects on chilling tolerance. Stomatal density (number of stomata per leaf area, SD) increased with increasing ratios of blue light whereas green light showed indications to decrease SD. | 27 |
| *^c^* Vanilla  *Vanilla planifolia Jacks.* | Five types of wavelengths were used: white light (W, 420 nm), red (R, 660 nm), blue (B, 460 nm), and a combination of blue and red LEDs (B:R, 1:1), also, a fluorescent light (Fl, 400–700 nm) control was included. Irradiation intensity of artificial light was set to 40 μmol m^−2^ s^−1^ at 16 h photoperiod. | In the multiplication phase, LEDs B:R stimulated the elongation of shoots and chlorophyll synthesis. In the rooting phase, LEDs B stimulated shoot elongation, the number of roots formed and the number of leaves per shoot. | 28 |
| *^c^* Sweet basil *Ocimum basilicum* L.  cv “Ceasar” | Plants were treated with blue with 450 nm peak (B), green with 520 nm peak (G), yellow/amber with 600 nm peak (Y), red with 600 nm peak (R), and far-red with 735  nm peak (Fr) LEDs at 100 μmol m^−2^ s^−1^ for exclusive B or R and for the sum of B and R (1:1 ratio – 50 μmol m^−2^ s^−1^ each). When a third wavelength was added (G, Y, or Fr), its contribution was 50 μmol m^−2^ s^−1^ (ratio1:1:1) giving a total fluence rate of 150 μmol m^−2^ s^−1^. The photoperiod was 12 h. | Basil plants grown under blue/red/yellow or blue/red/green wavelengths emit higher levels of a subset of monoterpenoid volatiles, while a blue/red/far-red treatment leads to higher levels of most sesquiterpenoid volatile molecules. Specific light treatments increase volatile content, mass, and antioxidant capacity. Narrow-bandwidth illumination can induce discrete suites of volatile classes that affect sensory quality in commercial herbs. | 29 |
| *^c^* *Rehmannia glutinosa* Libosch | Plants grown under conventional cool white fluorescent lamp (control), blue (450 nm peak) LED, or red (650 nm peak) LED at a 16 h photoperiod with 50 µmol m^−2^ s^−1^. | Blue or red LED treatments showed a significant increase in growth parameters compared with the cool white florescent light. In addition, the LED treatments increased the total phenol and flavonoid levels in leaf and root extracts. Furthermore, data on the total antioxidant capacity, reducing power potential, and DPPH radical scavenging capacity also revealed the enhancement of antioxidant capacity under both blue and red LED treatments. Especially, the blue LED treatment significantly increased the antioxidant enzyme activities in both the leaf and root, followed by the red LED treatment. Modulation in the spectral quality particularly by the blue LED induced the antioxidant defense line and was directly correlated with the enhancement of phytochemicals. | 30 |
| *^c^* Dill  *Anethum graveolens* L. | The share of orange and green light in the spectrum was constant and amounted to 10% for either colour. In the first combination (A, 70/10), there was 70% of red light and 10% of blue light. Other combinations had the following proportions: B 60/20, C 50/30, D 40/40 and E 30/50 of red and blue light. The PPFD was about 155 μmol m^-2^ s^-1^ given at a 16 h photoperiod. | Blue light inhibited the elongation growth as well as leaf area. It had positive influence on the accumulation of dry mass, glucose and fructose in the herb. In the combinations with higher percentage of red light the plants were characterised by higher content of essential oils, macronutrients and zinc. | 31 |
| *^c^* Mint  *Mentha piperita*, *Mentha spicata* and *Mentha longifolia*  Lentil  *Lens culinaris Medic*,  *^c^* Basil  *Ocimum basilicum* L.  *^d^* primula *Primula vulgaris*  *Huds.*,  *^d^* marigold *Calendula officinalis* L.,  *^d^* treasure flower  *Gazania splendens Moore* and  *^d^* Stock plant *Matthiola*  *incana* L. | Plants treated with 100% red, 100%blue, 70% red plus 30 % blue, or 100 % white LED lights at 500 μmol m^−2^ s^−1^ and 16 h photoperiod were compared with those grown under greenhouse conditions natural light at 235 – 1,800 μmol m^−2^ s^−1^. | 70/30 % red-blue LED light increased Mentha essential oil yield up to four times along with increases in plant photosynthesis and fresh weight compared with field condition. The red-blue LED incubator also led to a better growth of lentil and basil and to higher flower buds and less days to flowering. | 32 |
| *^d^* Chrysanthemum  *Chrysanthemum morifolium* Ramat | Plant treated with LED lights ranging from UV (280–400 nm) to far-red light (700–800 nm). Treatments with LED light combinations to obtain different spectral compositions were tested on rooted cuttings of 3 chrysanthemum genotypes (a pot chrysanthemum, a cut flower and a disbud chrysanthemum genotype) to assess the effect on shoot architecture. All treatments were at 60 μmol m^-2^ s^-1^ PAR for 19 h per day with fluorescent light as control. | Red light treatment generally showed increased bud outgrowth and increased average bud length while blue + far-red light treatment resulted in decreased bud outgrowth and bud length. Plant height, which increased under blue + far-red light treatments compared to red light treatment. | 33 |
| *^d^* Chrysanthemum *Chrysanthemum × morifolium* Ramat cv. ‘Riqietaohong’ | Plants treated with four different LED systems: red (R), blue (B), red : blue = 2:1 (RB) and blue : red = 2:1 (BR) provide at 50 ± 5 μmol m^-2^ s^-1^ PPFD for 16 h per day. | B light promoted stem and leaf development of seedlings but inhibited photosynthesis. The R light stimulated their rooting but inhibited the accumulation of biomass. RB improved transpiration RB was the best LED system for cut chrysanthemums. | 34 |
| *^d^* Chrysanthemum  *Chrysanthemum morifolium* ‘Zembla’ | Treatments comprised of RB (11 h of mixed red and blue [RB] light), RB + B (11 h of mixed RB light and then 4 h of supplemental B light), LRB + B (15 h of mixed RB light and then 4 h of supplemental B light) and RB + LB (11 h of mixed RB light and then 13 h of B light) by using LEDs maintained at 100 ± 5 μmol m^-2^ s^-1^. | Under mixed RB light, the net assimilation rate increased rapidly, then slightly decreased under B light, and finally dropped to negative values during darkness. Final stem length was the highest in plants grown under RB + LB, followed by LRB + B, RB + B and then RB treatment. The stem lengths under RB + B, LRB + B and RB + LB were 1.3, 1.5 and 1.7 times longer than that of RB treatment, respectively. | 35 |
| *^d^* Chrysanthemum  *Chrysanthemum morifolium* ‘Coral Charm’,  *^d^* Rose  *Rosa hybrida* ‘Scarlet’ and  *^d^* Campanula *Campabula portenschlagiana* ‘BluOne’ | The four light treatments were tested: (1) 40% Blue/60% Red, (2) 20% Blue/80% Red, (3) 100% Red, and (4) 100% White (Control) at 200 µmol m^−2^ s^−1^ for 16 h per day. | The plant height was smallest in 40% Blue/60% Red in roses and chrysanthemums, while the biomass was smallest in the White control in roses and in 100% Red in chrysanthemums. The total biomass was unaffected by the spectrum in campanulas, while the leaf area was smallest in the 40% Blue/60% Red treatment. In 100% Red curled leaves and other morphological abnormalities were observed. Increasing the blue to red ratio increased the stomatal conductance though net photosynthesis was unaffected. With higher blue light ratio all phenolic acids and flavonoids increased. | 36 |
| *^d^* Rose  *Rosa x hybrida* ‘Toril’ | HPS or LED light (blue; peaks at 402, 419, and 445 nm, and red/far-red; peaks at 663 and 737 nm) and one treatment with alternating HPS and LED light (three days each) with a PPFD of 100 – 120 µmol m^−2^ s^−1^ at 16 h photoperiod. | Stem elongation and leaf area were lower for plants grown under LED light while fresh and dry weight was unaffected. Plants subjected to alternating light regimes generally resembled LED treatment plants more than HPS plants. Leaf transmittance and reflectance were higher for leaves grown in HPS light. | 21 |
| *^d^* Sunflower *Helianthusannuus* cv. ‘Teddy Bear’ | LED (red 660 nm + blue 460 nm LED, 80:20 RB-LED; white LED, W-LED) vs. HPS lamps with a PPFD of 70–120 µmol m^−2^ s^−1^ at 16 h photoperiod. | Canopy surface area, dry and fresh weight, shoot length, internode number and length are lower in plants exposed to LEDs than HPS. | 37 |
| *^d^* Anthurium  *A. andreanum Lind.* and  *^d^* Moth orchids *Phalaenopsisis sp.* | Plants grown under white LEDs (460 and 560 nm), red LEDs (660 nm), blue LEDs (460 nm), the combination of blue and red LEDs (460 and 660 nm, respectively), and fluorescent lights, FLs (545–610 nm) as a control. All treatments were carried out at 25 μmol m^−2^ s^−1^ PPFD for 16 h per day. | For Anthurium, treatments with white LEDs, blue LEDs, and the combination of blue and red LEDs showed the greatest plantlet length and number of leaves. The FL and red LED treatments showed similar responses in promoting the formation of plantlets and their leaves. All shoots were rooted and the highest root number was induced in cultures incubated in FLs and blue LEDs with 6.6 and 6.0 roots, respectively. The lowest root number (1.5) was recorded in cultures incubated in red LEDs. Chlorophyll *a*, *b*, and total chlorophyll content was significantly higher in the blue LED treatment (0.692 mg g^−1^ fresh weight), while the lowest total chlorophyll content was found in the red LED and FL treatments with 0.327 and 0.375 mg g^−1^ fresh weight, respectively. For moth orchids, treatments with FLs, white LED and the combination of blue and red LEDs showed the greatest plantlet length and number of leaves. The white, red and blue LEDs showed similar responses in promoting the formation of plantlets and their leaves. All protocorms were rooted and had the same root number. Chlorophyll *a* content was significantly higher in the blue LED treatment (0.2813 mg g^−1^ fresh weight), while chlorophyll *b* content was higher in blue and the combination of blue and red LED treatments, with 0.1368 and 0.1468 mg g^−1^ fresh weight, respectively. Total chlorophyll (0.421875 mg g^−1^ fresh weight) was higher in blue LED. The lowest total chlorophyll content was found in FL treatments and white LEDs with 0.1810 and 0.2500 mg g^−1^ fresh weight, respectively. | 38 |
| *^d^* Carnation *Dianthus caryophyllus* cultivars ‘Green Beauty’ and ‘Purple Beauty’ | Plants grown under conventional cool white fluorescent lamp (control), blue (450 nm peak) LED, or red (650 nm peak) LED at a 16 h photoperiod with 50 µmol m^−2^ s^−1^. | Blue and red LED treatments resulted in a significant increase in growth, photosynthetic parameters, and nutrient content in comparison to the conventional cool white florescent lamp treatment. In addition, red LED treatment increased the activities of antioxidant enzymes and elemental contents in both cultivars. | 39 |
| *^d^* Petunia  *Petunia × hybrida* ‘Tidal wave’ | Plants grown under plastic film transmitting (+FR) and not transmitting far-red light (−FR) in combination with R (660 nm peak) and B (450 nm peak) LEDs given as supplementary light placed underneath the films. The objective of the study was to test the effect of R and B lights on morphology and flowering in a FR-deficient greenhouse environment compared with an environment with FR light. LED lights were provided at 50 μmol m^-2^ s^-1^ for 16 h per day. HPS lamps were used as regular top light above the plastic films and the PAR light was 180 μmol m^-2^ s^-1^ in the +FR control treatment as well as the –FR treatment and 100 μmol m^-2^ s^-1^ in the LED treatments to provide similar total amount of supplementary light in all treatments. | R light reduced shoot elongation and resulted in more compact plants in both seasons and the combination of –FR and R light resulted in the most compact plants without much delay in flowering. In early spring when the natural irradiance was low (1.35 mol m^−2^ h^−1^), B light promoted stem elongation, caused a more upright shoot orientation, increased plant height and promoted flowering compared to control and R light, whereas in late spring, when the natural irradiance was higher (2.33 mol m^−2^ h^−1^), the effect of B light in a FR-deficient environment was not significantly different from R light in the response to stem elongation and flowering. | 40 |
| *^d^* Balloon flower *Platycodon grandiflorum* (Jacq.) A. DC. | Plants grown under LEDs at a PPFD of 50 50 µmol m^−2^ s^−1^ at 12 h photoperiod using blue (460 nm peak) light (B), 75% blue + 25% red (658 nm peak) light (BR31), 50% blue + 50% red light (BR11), 25% blue + 75% red light (BR13) and red light (R). Fluorescent white lamps (FL) were used as a control. | Treatment B induced larger leaf area, leaf thickness and dry mass of whole plant and higher leaf number than treatment R or FL. Plantlets from treatment R had the highest specific leaf mass, soluble sugar and sucrose content. Chlorophyll and L-ascorbic acid content was higher in leaves grown under LED variants than in those grown under FL. The indoleacetic acid content in B- and BR31-treated leaves was higher than that in the R-treated leaves. In addition, B and BR31 induced large and nearly rectangular palisade parenchyma cells, while the palisade parenchyma cells were elliptical in shape and small in the FL, BR11 and BR13 treatments, or irregular in the R treatment. | 41 |
| *^d^* Perilla  *Perilla frutescens* var. *acuta* Kudo | Grown at 140 ± 20 μmol m^−2^ s^−1^ PPF at 12 h photoperiod provided by either cool white fluorescent lamps (FL, control), white (W) light emitting diodes (LEDs), or an 8:1:1 mixture of red, blue and white (RBW) LEDs. | Plant height increased in the FL as compared to the W and RBW LEDs treatments. The RBW LEDs treatment promoted vegetative growth of the shoot and root. Chlorophyll fluorescence (Fv/Fm) was not significantly affected by the light source. Total anthocyanin concentration per leaf was higher in the RBW LEDs treatment than the other treatments. | 42 |
| Medicinal plant  *Crepidiastrum denticulatum* | Plants treated with various periods of irradiation and ratios of far-red (735 nm peak) (FR) LED light combined with red (660 nm peak) (R) and blue (440 nm peak) (B) LED lights. The ratio of R to B LEDs was set at 8:2 (R8B2), and the ratio of R to FR LEDs was adjusted to 0.7, 1.2, 4.1, and 8.6 R8B2 (without FR LEDs) and commercial LEDs were used as control I and II, respectively. The plants were cultivated under these light treatments, and some of the control plants were subjected to four different R/FR ratios for 30 min before the end of the light period (EOL). PPFD of all light treatments, including the controls, was 130 ± 5 µmol m^−2^ s^−1^ given at 16 h photoperiod. | Shoot fresh and dry weight, leaf area, leaf length, and leaf width under R/FR ratios of 0.7 and 1.2 were 1.8- to 2.4-times higher than those of the control plants. Continuous R/FR 0.7 and 1.2 irradiation reduced the total phenolic content per dry weight compared to the control, although this effect was not significant. The total phenolic content per plant under R/FR 0.7 and 1.2 increased more than 2-fold, and the shoot fresh weight increased 1.7- and 1.6-fold, compared to control I. Individual phenolic compound levels exhibited the same trends as total phenolic content. Under R/FR 0.7 and 1.2, chlorogenic acid, caffeic acid, and chicoric acid levels per shoot increased 1.3- to 1.8-fold compared to control I FR EOL treatments did not have significant effects on growth or the contents of bioactive compounds | 43 |
| White beech mushroom *Hypsizygus marmoreus* | Plants grown under blue (475 nm), green (525 nm), yellow (590 nm), or red (660 nm) LEDs using fluorescent lights and darkness as controls. Lights were continuous and provided at below 150 lux. | The diameter and thickness of the pileus and length of stipes in samples subjected to blue LED treatment were similar to those of subjected to fluorescent light (control), and the lengths of the stipes were highest in response to treatment with the red LED and darkness. The commercial  yields of plants subjected to blue and green LED treatment were similar to those of the control. | 44 |
| Dropwort *Oenanthe stolonifera* | Plants grown under three monochromatic LEDs: red (R; 654 nm), blue (B; 456 nm), and green (G; 518 nm), thirteen combinations of R and B (R:B = 9:1, 8:2, 7:3, and 6:4), RB with G (R:G:B = 9:1:0, 8:1:1, 7:1:2, and 6:1:3), and RB with white (W) (R:W:B = 8:2:0, 8:1:1, 7:2:1, 7:1:2, and 6:2:2) and fluorescent lamps (control) at 171 ± 5 μmol m^-2^ s^-1^ of PPFD for 12 h per day. | R LEDs improved growth characteristics, including plant height and fresh and dry weights of shoots. Combined LEDs with 70-80% red wavelength resulted in the highest values in fresh weight. The B LED treatment resulted in the highest total phenolic and anthocyanin content in dropwort leaves, which increased in the combined LEDs treatments as the proportion of blue wavelength increased. Further, the RWB treatments, regardless of the ratio, resulted in higher anthocyanin contents than that in the RB and RGB treatments. Persicarin content was also significantly higher in the B treatment than in the R and G treatments. However, compared to the RB treatments, persicarin content in the RGB and RWB treatments was decreased, on average, by 72 and 64%, respectively. | 45 |
| Figwort  *Scrophularia takesimensis* Nakai | Plant cultures were treated with 45 μmol m^-2^ s^-1^ blue or red LEDs and white fluorescence lights (WFL) (control) for 16 h photoperiod | LED gave better shoot growth followed by WFL and blue LED. However, red LED treatment decreased the number of roots and induced callus at the base of shoot. The greatest frequency of flower induction (96.8 %) was obtained under blue LED. | 46 |
| Oak tree  *Quercus ithaburensis* var. macrolepis | Seedlings were pre-cultivated under five different LED light qualities: (1) Fluorescent (FL) as control light (2) L20AP67 (high in green and moderate in far-red), (3) AP673L (high in green and red), (4) G2 (highest in red and far-red), AP67 (high in blue, red, and far-red), and (5) NS1 (highest in blue and green and lowest in far-red) LEDs provided at 150 ± 10 µmol m^−2^ s^−1^ PPFD for 17 h per day. | AP67 and AP673L triggered higher leaf formation, while L20AP67 positively affected seedling shoot development. NS1 and AP67 LED pre-cultivated seedlings showed significantly higher root fibrosity than those of FL light. Furthermore, NS1 and AP673L LEDs induced fourfold increase on seedling root dry weight than FL light. LED pre-cultivated seedlings showed higher survival and faster growth indicating better adaptation even under natural light conditions. | 47 |
| Oriental plane tree  *Platanus orientalis* L. | LED (33% red + 33% green + 33% blue and 66% red + 33% blue) vs. white fluorescent lamps with a PPFD of 200 µmol m^−2^ s^−1^ at 12 h photoperiod. | LED grown plants have lower height, biomass and leaf area but higher leaf lamina thickness and bigger stomata size. LED grown plants produced lower isoprene. | 20 |
| Ice plant  *Mesembryanthemum crystallinum* | Plants were grown under different  Red (670 nm peak):blue (465 nm peak) LED ratios: (1) 100:0 (0B); (2) 90:10 (10B); (3) 80:20 (20B); (4) 70:30 (30B); (5) 50:50 (50B); and (6)100:0 (100B) for a 16 h photoperiod at 350 μmol m^−2^ s^−1^ PPFD | Plants grown under 10B had the highest shoot and root biomass and shoot/root ratio while those grown under 0B had the lowest values. Compared to plants grown under 0B condition, all other plants had similar but higher total chlorophyll (Chl) and carotenoids (Car) contents and higher Chl *a*/*b* ratios. | 48 |
| Arabidopsis  *Arabidopsis thaliana* (L.) Heynh. | Plants were grown under a combination of fluorescent lamps (Osram L36W/830 und L36W/840, Osram) and two LED systems: ldv (type L18SP673 L, Valoya) and the custom-made LED system ldr (Roschwege)  that combined LEDs with the light colour warm white 3.000K with 660 nm LEDs of moderate and 730 nm wavelength LEDs  of low intensity. The three light sources were set to an intensity of 180 µmol m^–2^ s^–1^ maintained at a 16 h photoperiod. | All genotypes had significantly higher rosette dry weight and seed mass and developed faster under LED lights than under fluorescent lamps. Chlorophyll content, photosynthetic complex accumulation and light response curves of chlorophyll fluorescence parameters were indistinguishable under LED and fluorescent light.  Principal component analysis of leaf metabolite concentrations revealed that the effect of a change from fluorescent light to LED light was small compared with the diurnal effect, which explains 74% of the variance and the age effect during vegetative growth (12%). | 49 |

Note: LED represents light-emitting diodes; HPS represents high-pressure sodium lamps; PAR represents photosynthetically active radiation; PPFD represents photosynthetic photon flux density; *a* represents green crop plants; *b* represents fruits; *c* represents herbs; and *d* represents ornamentals.

**References**

1 Amoozgar, A., Mohammad, A. & Sabzalian, M. R. Impact of light-emitting diode irradiation on photosynthesis, phytochemical composition and mineral element content of lettuce cv. Grizzly. *Photosynthetica* **55**, 85-95, doi:10.1007/s11099-016-0216-8 (2017).

2 Chen, X.-l. *et al.* Growth and nutritional properties of lettuce affected by different alternating intervals of red and blue LED irradiation. *Sci Hortic* **223**, 44-52 (2017).

3 Długosz-Grochowska, O., Wojciechowska, R., Kruczek, M. & Habela, A. Supplemental lighting with LEDs improves the biochemical composition of two *Valerianella locusta* (L.) cultivars. *Hortic Environ Biotechnol* **58**, 441-449, doi:10.1007/s13580-017-0300-4 (2017).

4 Bantis, F., Ouzounis, T. & Radoglou, K. Artificial LED lighting enhances growth characteristics and total phenolic content of Ocimum basilicum, but variably affects transplant success. *Sci Hortic* **198**, 277-283, doi:<http://dx.doi.org/10.1016/j.scienta.2015.11.014> (2016).

5 Chen, Z. *et al.* Application and optimization of organic-inorganic hybrid monolithic capillary electrochromatography for in vivo cefdinir determination with microdialysis. *J Sep Sci* **39**, 440-449, doi:10.1002/jssc.201500817 (2016).

6 Kang, W. H., Park, J. S., Park, K. S. & Son, J. E. Leaf photosynthetic rate, growth, and morphology of lettuce under different fractions of red, blue, and green light from light-emitting diodes (LEDs). *Hortic Environ Biotechnol* **57**, 573-579, doi:10.1007/s13580-016-0093-x (2016).

7 Son, K.-H., Jeon, J.-M. & Oh, M.-M. Application of supplementary white and pulsed light-emitting diodes to lettuce grown in a plant factory with artificial lighting. *Hortic Environ Biotechnol* **57**, 560-572, doi:10.1007/s13580-016-0068-y (2016).

8 Jishi, T., Matsuda, R. & Fujiwara, K. A kinetic model for estimating net photosynthetic rates of cos lettuce leaves under pulsed light. *Photosynth Res* **124**, 107-116 (2015).

9 Lee, M.-J., Park, S.-Y. & Oh, M.-M. Growth and cell division of lettuce plants under various ratios of red to far-red light-emitting diodes. *Hortic Environ Biotechnol* **56**, 186-194, doi:10.1007/s13580-015-0130-1 (2015).

10 Son, K.-H. & Oh, M.-M. Growth, photosynthetic and antioxidant parameters of two lettuce cultivars as affected by red, green, and blue light-emitting diodes. *Hortic Environ Biotechnol* **56**, 639-653, doi:10.1007/s13580-015-1064-3 (2015).

11 Wojciechowskaa, R., Długosz-Grochowskaa, O., Kołtona, A. & Zupnik, M. Effects of LED supplemental lighting on yield and some quality parameters of lamb’s lettuce grown in two winter cycles. *Sci Hortic* **187**, 80-86 (2015).

12 Chang, C.-L. & Chang, K.-P. The growth response of leaf lettuce at different stages to multiplewavelength-band light-emitting diode lighting. *Sci Hortic* **179**, 78-84 (2014).

13 Vaštakaitė, V. *et al.* Pulsed light-emitting diodes for a higher phytochemical level in microgreens. *J Agr Food Chem* **65**, 6529-6534, doi:10.1021/acs.jafc.7b01214 (2017).

14 Rabara, R. C., Behrman, G., Timbol, T. & Rushton, P. J. Effect of spectral quality of monochromatic LED lights on the growth of artichoke seedlings. *Front Plant Sci* **8**, 190, doi:10.3389/fpls.2017.00190 (2017).

15 Avercheva, O. V. *et al.* Optimizing LED lighting for space plant growth unit: Joint effects of photon flux density, red to white ratios and intermittent light pulses. *Life Sci Space Res (Amst)* **11**, 29-42, doi:10.1016/j.lssr.2016.12.001 (2016).

16 Brazaityte, A. *et al.* The effects of LED illumination spectra and intensity on carotenoid content in Brassicaceae microgreens. *Food Chem* **173**, 600-606 (2015).

17 Li, H., Tang, C. & Xu, Z. The effects of different light qualities on rapeseed (*Brassica napus* L.) plantlet growth and morphogenesis *in vitro*. *Sci Hortic* **150**, 117-124 (2013).

18 Simlat, M. *et al.* The effect of light quality on seed germination, seedling growth andselected biochemical properties of *Stevia rebaudiana* Bertoni. *Sci Hortic* **211**, 295-304 (2016).

19 Lanoue, J., Leonardos, E. D., Ma, X. & Grodzinski, B. The effect of spectral quality on daily patterns of gas exchange, biomass gain, and water-use-efficiency in tomatoes and lisianthus: An assessment of whole plant measurements. *Front Plant Sci* **8**, 1076, doi:10.3389/fpls.2017.01076 (2017).

20 Arena, C. *et al.* The effect of light quality on growth, photosynthesis, leaf anatomy and volatile isoprenoids of a monoterpene-emitting herbaceous species (*Solanum lycopersicum* L.) and an isoprene-emitting tree (*Platanus orientalis* L.). *Environ Exp Bot* **130**, 122-132 (2016).

21 Bergstrand, K.-J., Mortensen, L. M., Suthaparan, A. & Gislerød, H. R. Acclimatisation of greenhouse crops to differing light quality. *Sci Hortic* **204**, 1-7 (2016).

22 Matsuda, R., Yamano, T., Murakami, K. & Fujiwara, K. Effects of spectral distribution and photosynthetic photon flux densityfor overnight LED light irradiation on tomato seedling growth and leaf injury. *Sci Hortic* **198**, 363-369 (2016).

23 Khoshimkhujaev, B., Kwon, J. K., Park, K. S., Choi, H. G. & Lee, S. Y. Effect of monochromatic UV-A LED irradiation on the growth of tomato seedlings. *Hortic Environ Biotechnol* **55**, 287-292 (2014).

24 Yoshida, H., Mizuta, D., Fukuda, N., Hikosaka, S. & Goto, E. Effects of varying light quality from single-peak blue and red light-emitting diodes during nursery period on flowering, photosynthesis, growth, and fruit yield of everbearing strawberry. *Plant Biotechnol* **33**, 267-276 (2016).

25 Choi, H. G., Moon, B. Y. & Kang, N. J. Effects of LED light on the production of strawberry during cultivationin a plastic greenhouse and in a growth chamber. *Sci Hortic* **189**, 22-31 (2015).

26 Hung, C. D. *et al.* Growth and morphogenesis of encapsulated strawberry shoot tips under mixed LEDs. *Sci Hortic* **194**, 194-200 (2015).

27 Jensen, N. B., Clausen, M. R. & Kjaer, K. H. Spectral quality of supplemental LED grow light permanently alters stomatal functioning and chilling tolerance in basil (*Ocimum basilicum* L.). *Sci Hortic* **227**, 38-47 (2018).

28 Ramírez-Mosqueda, M. A., Iglesias-Andreu, L. G. & Luna-Sánchez, I. J. Light quality affects growth and development of *in vitro* plantlet of *Vanilla planifolia* Jacks. *S Afr J Bot* **109**, 288-293 (2017).

29 Carvalho, S. D., Schwieterman, M. L., Abrahan, C. E., Colquhoun, T. A. & Folta, K. M. Light quality dependent changes in morphology, antioxidant capacity, and volatile production in sweet basil (Ocimum basilicum). *Front Plant Sci* **7**, 1328, doi:10.3389/fpls.2016.01328 (2016).

30 Manivannan, A., Soundararajan, P., Halimah, N., Ko, C. H. & Jeong, B. R. Blue LED light enhances growth, phytochemical contents, and antioxidant enzyme activities of *Rehmannia glutinosa* cultured *in vitro*. *Hortic Environ Biotechnol* **56**, 105-113 (2015).

31 Frąszczak, B., Gąsecka, M., Golcz, A. & Zawirska-Wojtasiak, R. The effect of radiation of LED modules on the growth of dill (*Anethum graveolens* L.). *Open Life Sci* **11**, 61-70 (2016).

32 Sabzalian, M. R. *et al.* High performance of vegetables, flowers, and medicinal plants in a red-blue LED incubator for indoor plant production. *Agron Sustain Dev* **34**, 879-886, doi:10.1007/s13593-014-0209-6 (2014).

33 Dierck, R., Dhooghe, E., Huylenbroeck, J. V., Van Der Straeten, D. & De Keyser, E. Light quality regulates plant architecture in different genotypes of *Chrysanthemum morifolium* Ramat. *Sci Hortic* **218**, 177-186 (2017).

34 Hong, Y., Huang, H. & Dai, S. An in vivo study of the best light emitting diode (LED) systems for cut chrysanthemums. *Open Life Sci* **10**, 310-321 (2015).

35 Jeong, S. W., Hogewoning, S. W. & van Ieperen, W. Responses of supplemental blue light on flowering and stem extension growth of cut chrysanthemum. *Sci Hortic* **165**, 69-74 (2014).

36 Ouzounis, T., Frette, X., Rosenqvist, E. & Ottosen, C. O. Spectral effects of supplementary lighting on the secondary metabolites in roses, chrysanthemums, and campanulas. *J Plant Physiol* **171**, 1491-1499, doi:10.1016/j.jplph.2014.06.012 (2014).

37 Alsanius, B. W. *et al.* Ornamental flowers in new light: Artificial lighting shapes themicrobial phyllosphere community structure of greenhouse grownsunflowers (*Helianthus annuus* L.). *Sci Hortic* **216**, 234-247 (2017).

38 Bello‐Bello JJ, Pérez‐Sato JA, Cruz‐Cruz CA, *et al.* Light‐emitting diodes: Progress in Plant Micropropagation, Chlorophyll, Prof. Eduardo Jacob-Lopes (Ed.), InTech, doi: 10.5772/67913. Available from: https://www.intechopen.com/books/chlorophyll/light-emitting-diodes-progress-in-plant-micropropagation (2017).

39 Manivannan, A. *et al.* Blue and red light-emitting diodes improve the growth and physiology of *in vitro*-grown carnations ‘green beauty’ and ‘purple beauty’. *Hortic Environ Biotechnol* **58**, 12-20 (2017).

40 Gautam, P., Terfa, M. T., Olsen, J. E. & Torre, S. Red and blue light effects on morphology and flowering of *Petunia × hybrida*. *Sci Hortic* **184**, 171-178 (2015).

41 Liu, M. *et al.* Evaluation of leaf morphology, structure and biochemical substanceof balloon flower (*Platycodon grandiflorum* (Jacq.) A. DC.) plantlets *in vitro* under different light spectra. *Sci Hortic* **174**, 112-118 (2014).

42 Park, Y. G., Oh, H. J. & Jeong, B. R. Growth and anthocyanin concentration of *Perilla frutescens* var. *acuta* Kudo as affected by light source and DIF under controlled environment. *Hortic Environ Biotechnol* **54**, 103-108 (2013).

43 Bae, J.-H., Park, S.-Y. & Oh, M.-M. Supplemental irradiation with far-red light-emitting diodes improves growth and phenolic contents in *Crepidiastrum denticulatum* in a plant factory with artificial lighting. *Hortic Environ Biotechnol* **58**, 357-366 (2017).

44 Jang, M. J., Lee, Y. H., Ju, Y. C., Kim, S. M. & Koo, H. M. Effect of Color of Light Emitting Diode on Development of Fruit Body in Hypsizygus marmoreus. *Mycobiology* **41**, 63-66, doi:10.5941/MYCO.2013.41.1.63 (2013).

45 Jeon, Y.-M., Son, K.-H., Kim, S.-M. & Oh, H. J. Growth and bioactive compounds as affected by irradiation with various spectrum of light-emitting diode lights in dropwort. *Hortic Environ Biotechnol* **58**, 467-478 (2017).

46 Jeong, B. R. & Sivanesan, I. Direct adventitious shoot regeneration, *in vitro* flowering, fruiting, secondary metabolite content and antioxidant activity of *Scrophularia takesimensis* Nakai. *Plant Cell and Tissue Organ Cult* **123**, 607-618 (2015).

47 Smirnakou, S., Ouzounis, T. & Radoglou, K. M. Continuous spectrum LEDs promote seedling quality traits and performance of Quercus ithaburensis var. macrolepis. *Front Plant Sci* **8**, 188, doi:10.3389/fpls.2017.00188 (2017).

48 He, J., Qin, L., Chong, E. L., Choong, T. W. & Lee, S. K. Plant growth and photosynthetic characteristics of Mesembryanthemum crystallinum grown aeroponically under different blue- and red-LEDs. *Front Plant Sci* **8**, 361, doi:10.3389/fpls.2017.00361 (2017).

49 Köhl, K., Tohge, T. & Schöttler, M. A. Performance of *Arabidopsis thaliana* under different light qualities: comparison of light-emitting diodes to fluorescent lamp. *Funct Plant Biol* **44**, 727-738 (2017).
